# Cardiac Troponins for the Clinical Management of Patients with Claudication and without Cardiac Symptoms

**DOI:** 10.3390/jcm11247287

**Published:** 2022-12-08

**Authors:** Dimitrios Mouselimis, Saskia Hagstotz, Michael Lichtenberg, Konstantinos P. Donas, Ulrike Heinrich, Konstantinos Avranas, Zisis Dimitriadis, Erwin Blessing, Ralf Langhoff, Norbert Frey, Hugo A. Katus, Grigorios Korosoglou

**Affiliations:** 1 Cardiology, Vascular Medicine & Pneumology, GRN Hospital Weinheim, Roentgentrasse 1, 69469 Weinheim, Germany; 2 Vascular Center Klinikum Arnsberg, 59821 Arnsberg, Germany; 3 Department of Vascular and Endovascular Surgery, Asclepios Clinic Langen, 63225 Langen, Germany; 4 Practice for Vascular Medicine and Gastroenterology, 69469 Weinheim, Germany; 5 Department of Cardiology, Asclepios Clinic Langen, 63225 Langen, Germany; 6 Department of Cardiology, University Hospital Frankfurt, 60590 Frankfurt, Germany; 7 SRH Hospital, Department of Internal Medicine, 76307 Karlsbad, Germany; 8 Department of Angiology, Sankt-Gertrauden-Krankenhaus, 10713 Berlin, Germany; 9 Cardiology, Angiology & Pneumology, University Hospital Heidelberg, 69120 Heidelberg, Germany

**Keywords:** peripheral artery disease, cardiac troponins, coronary artery disease, acute myocardial injury, outcomes, claudication

## Abstract

Many patients with peripheral arterial disease (PAD) exhibit undiagnosed obstructive coronary artery disease. We aim to identify the patients with lifestyle limiting claudication due to PAD and without cardiac symptoms, requiring coronary revascularization based on high-sensitive troponin T (hsTnT) values. We assessed hsTnT in consecutive patients referred for elective endovascular treatment due to claudication [Rutherford categories (RC) 2 & 3] between January 2018 and December 2021. Diagnostic work-up by non-invasive imaging and, if required, cardiac catheterization was performed according to clinical data, ECG findings and baseline hsTnT. The occurrence of cardiac death, myocardial infarction or urgent revascularization during follow-up was the primary endpoint. Of 346 patients, 14 (4.0%) exhibited elevated hsTnT ≥ 14 ng/L, including 7 (2.0%) with acute myocardial injury by serial hsTnT sampling. Coronary revascularization by percutaneous coronary intervention was necessary in 6 of 332 (1.5%) patients with normal versus nine of 14 (64.3%) patients with elevated hsTnT (*p* < 0.001). During 2.4 ± 1.4 years of follow-up, 20 of 286 (7.0%) patients with normal versus four of 13 (30.8%) with elevated hsTnT at baseline reached the composite primary endpoint (*p* = 0.03 by log-rank test). In conclusion, elevated troponins in cardiac asymptomatic patients with claudication modify subsequent cardiac management and may increase the need for closer surveillance and more aggressive conservative management in polyvascular disease.

## 1. Introduction

Peripheral artery disease (PAD) currently affects more than 200 million individuals worldwide [1], and its incidence is expected to further rise in the coming decades. Although the prevalence of PAD is higher in countries with higher income, the majority of PAD patients are found in countries with middle to lower income. Race also plays a significant role, with the Black race being more prone to suffer from PAD, whilst people of Asian origin have the lowest prevalence [2]. Despite the improvement of pharmacologic and endovascular treatment options, these patients still endure high morbidity and mortality rates, mainly triggered by cardiac events [3,4,5,6]. Based on previous reports, many patients with PAD exhibit undiagnosed obstructive coronary artery disease (CAD) [7].

Cardiac troponins are well-established markers, predicting mortality and adverse cardiovascular events in patients with stable CAD [8]. In addition, high troponin levels within the upper limit of normal are associated with increased cardiovascular risk in the general population, independent of conventional atherogenic risk factors [9]. Patients with PAD are *per se* at increased cardiovascular risk [10,11]. However, the usefulness of cardiac troponins to guide clinical management of patients presenting with symptomatic PAD due to claudication remains unclear. In addition, patients with claudication are limited in terms of physical activity, so that cardiac symptoms such as dyspnea or angina may not be detectable during daily activities or treadmill testing. However, current guidelines do not routinely recommend non-invasive cardiac work-up in the absence of cardiac symptoms in patients with PAD [12,13].

Elevated levels of cardiac troponins are present not only in myocardial necrosis, but also in other chronic or acute diseases [14]. Renal failure causing a reduced repletion of troponins and sepsis increasing the permeability of all cells including the cardiomyocytes may also lead to an elevation of the cardiac enzymes without the presence of CAD [15,16]. Excessive cardiac stress related to sports, chronic heart failure and other stressful conditions for the heart muscle may also lead to myocardial injury, which also unleashes high amounts of troponins in the blood [15]. Considering that most patients with PAD have, apart from their polyvascular disease, a multitude of comorbidities, it is conceivable that these cause an increase of the troponin levels as a result of myocardial stress [10,11]. Therefore, a proper strategy for the further management of the suspected CAD in patients with PAD appears to be crucial.

In our study, we sought to investigate the ability of high-sensitive troponin T (hsTnT) to guide clinical management in cardiac asymptomatic patients with claudication referred for elective endovascular treatment. In addition, we investigated the influence of increased troponin levels on subsequent cardiac events during follow-up.

## 2. Materials and Methods

### 2.1. Study Design and Patient Population 

We performed a single center observational study, aiming at assessing the demographic, clinical and follow-up data in patients without cardiac symptoms who were referred to our department for elective endovascular treatment of PAD due to claudication (RC 2 and 3) between January 2018 and December 2021. 

Traditional cardiovascular risk factors, including arterial hypertension, hyperlipidemia, current or prior smoking, diabetes mellitus and family history of CAD as well as history of CAD, myocardial infraction, or prior percutaneous coronary interventions (PCI) were recorded. PAD status was classified based on the Rutherford categories (RC). Laboratory markers, including hsTnT, hemoglobin, serum creatinine and the estimated glomerular filtration rate [GFR] and HbA1c were analyzed. 

Patients who presented to our emergency department with acute or subacute limb ischemia, with rest pain or with wound healing disorders RC 4-6, as well as patients with chronic kidney disease with GFR < 30 mL/min or chronic hemodialysis or platelet count < 100 × 10^9^/L, anemia with Hb < 10.0 g/dL, known significant liver disease (e.g., acute clinical hepatitis, chronic active hepatitis, cirrhosis, or alanine transaminase > 3ULN), childbearing potential without proper contraceptive measures, pregnancy or breast feeding and severe concomitant condition or disease (e.g., life expectancy < 6 months secondary to cancer, advanced liver disease or dementia) were excluded from analysis. Approval was obtained from the local ethics committee (S-100/2017) of the University of Heidelberg and all patients provided written informed consent. Patients or the public were not involved in the design, conduct, reporting or dissemination plans of the research.

### 2.2. Cardiac Troponins 

Blood samples were drawn from a peripheral vein and hsTnT was measured using the new quantitative electrochemiluminescence immunoassay (Cobas 411, Roche Diagnostics, Mannheim, Germany), as described previously [17]. A concentration of 14 ng/L has been identified as the 99th percentile of a healthy reference population with a coefficient of variability of <10% [18]. Chronic myocardial injury was defined as increased but stable troponin levels in terms of a rise/fall ≤20% in serial measurements, and acute myocardial injury was defined as the rise or fall of troponin >20% in serial measurements [19,20].

### 2.3. Peripheral Endovascular Procedures

All procedures were performed by experienced endovascular specialists, board certified for the endovascular treatment of PAD by the German Societies of Cardiology and Vascular Medicine. Iliac lesions were treated using standard balloon angioplasty followed by self- or balloon expanding bare metal stent implantation. Femoropopliteal lesions were treated using standard balloon angioplasty for pre-dilatation, followed by drug coated balloon (DCB) angioplasty and stent placement if deemed necessary. Atherectomy was performed prior to balloon angioplasty in moderate to severely calcified femoropopliteal lesions [21].

### 2.4. Cardiac Testing and Catheterization

Clinical assessment, electrocardiography (ECG) and cardiac troponin testing were performed in all patients. In patients with increased hsTnT ≥ 14 ng/L, an echocardiography was performed. In addition, serial troponin sampling occurred 1–3 h after the first test to detect the presence or absence of acute myocardial injury. Based on the electrocardiogram, echocardiography and serial troponin sampling, patients underwent non-invasive cardiac testing, including stress-echocardiography, stress cardiac magnetic resonance or cardiac computed tomography angiography (CCTA), or were scheduled directly for invasive cardiac catheterization.

In patients who underwent cardiac catheterization, standard angiographic projections were acquired. In cases of stenosis > 90% (diameter stenosis by visual estimation) in arteries with vessel diameter >2.5 mm, a PCI was performed. In cases of diameter stenosis between 40% and 90%, a PCI was performed only if a positive stress test by stress echocardiography or cardiac magnetic resonance was available. If stress testing was not available, FFR measures were performed using the Quantien™ System (Abbott, Santa Clara, CA, USA) and a coronary pressure wire (PressureWire™ X; Abbott), as described previously [22]. FFR values of ≤0.80 were considered as indicative of myocardial ischemia.

PCI within the first 90 days was considered as early revascularization, triggered by the diagnostic cardiac work-up. In patients with normal hsTnT, a further cardiac work-up was performed, depending on decisions made by the treating physicians and independent of the present study.

### 2.5. Endpoints of Our Study 

Follow-up was conducted using a structured clinical telephone interview with the patients or their family relatives. The follow-up was performed by a trained research assistant (SH) and was supervised by experienced physicians. Questions collected aimed at identifying the occurrence of cardiovascular events such as cardiac death, myocardial infarction, and revascularization during follow-up, which was defined as our composite primary endpoint. Repeated revascularizations were defined as revascularization performed >90 days after the initial presentation of the patients. For all patients, a thorough review of the medical records was performed to identify coronary revascularizations, cardiovascular events, and possible causes of death. 

### 2.6. Statistical Analysis

Analysis was performed using the commercially available software MedCalc 18.5 (MedCalc software, Mariakerke, Belgium). Continuous normally distributed variables were expressed as mean ± standard deviation, whereas non-normally distributed variables like the hsTnT were also reported as medians with interquartile range (IQR). Categorical variables were reported as proportions. Categorical data were compared using c² tests or by the Fischer’s exact test. Patients were separated based on their baseline hsTnT values in those with normal hsTnT <14 ng/L and patients with elevated hsTnT ≥ 14 ng/L. In addition, analysis was performed using hsTnT quartiles. A Kaplan-Meier analysis was used for the calculation of the primary endpoint during follow-up. Multivariable logistic regression models were used to assess the value of hsTnT of the prediction of short-term coronary revascularization in patients with claudication. The ANOVA test was used for comparing three or more normally distributed groups with the Scheffé test for post-hoc analysis [23]. Differences were considered statistically significant at *p* < 0.05.

## 3. Results

### 3.1. Demographic Data and Peripheral Endovascular Procedures 

Between January 2018 and December 2021, 346 patients were referred to our department for elective endovascular treatment due to claudication after being diagnosed by referring angiologists, cardiologists or within our out-patient center. Fourteen out of 346 patients (4.0%) exhibited elevated baseline hsTnT values (≥14 ng/L). Patient characteristics based on baseline hsTnT are provided in Table 1. Patients with elevated hsTnT were more frequently male (59.5%), had more atherogenic risk factors, and often a history of CAD and previous infarction. 

### 3.2. Peripheral Endovascular Procedures

Interventions were performed in the iliac, common femoral artery (CFA), femoropopliteal segments, and below-the-knee (BTK) arteries in 134 (38.7%), 20 (5.7%), 244 (70.5%) and 12 (3.4%) cases, respectively. Endovascular treatment was successful in 346 (99.1%) patients, resulting in <30% residual stenosis of the treated segments. Lesions were treated using plain old balloon angioplasty (POBA), drug coated balloons (DCBs), scoring balloon and atherectomy in 169 (48.8%), 257 (74.3%), 28 (8.1%) and 118 (34.1%) cases, respectively. Stent implantation was performed in 103 (76.8%) iliac, 1 (5.0%) CFA, 75 (30.7%) femoropopliteal and 3 (25.0%) BTK lesions, respectively.

### 3.3. Predictors of Elev#ated hsTnT 

A history of CAD and myocardial infarction were both strong predictors of elevated hsTnT in PAD patients, independent of age, gender and estimated GFR [Table 2, HR of 5.8 (95%CI = 1.7–19.7) and 33.2 (95%CI = 9.0–122.5), respectively, *p* < 0.01 for both].

### 3.4. Cardiac Management by hsTnT and Subsequent Coronary Revascularization

**Figure 1:**
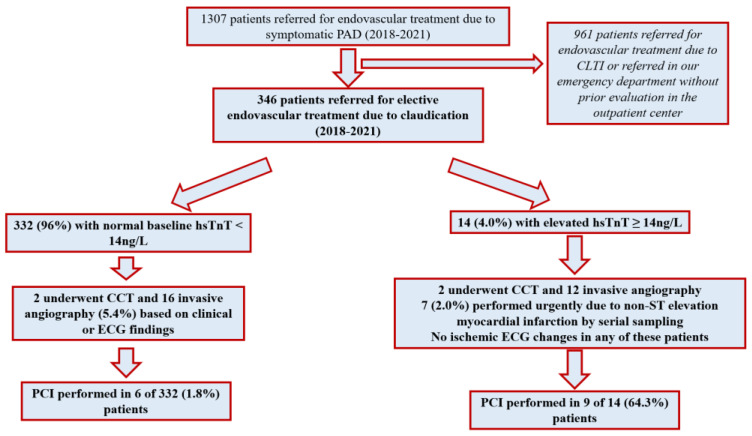
Flow chart of our study, including inclusion of patients with PAD and further clinical management regarding cardiac work-up and coronary revascularization.

Of 332 patients with normal hsTnT, 18(5.4%) underwent CCTA (*n* = 2) or cardiac catheterization (*n* = 16) due to the following reasons: ECG or wall motion abnormalities by echocardiography (*n* = 12) or self-reported clinical symptoms, which had not been reported during the initial diagnostic work-up for PAD (*n* = 4) or upon decision of the treating physicians (*n* = 2). PCI was performed in 6 of 332(1.8%) patients. PCI was performed in one coronary artery in four of the patients and in two out of three arteries in the remaining two patients (mean number of stents 2.0 ± 1.1) (Figure 1).

Of 14 patients with elevated hsTnT, all underwent CCTA (*n* = 2) or cardiac catheterization (*n* = 12). Cardiac catheterization was performed urgently prior to the endovascular procedure for PAD, as scheduled upon referral in seven (50%) patients due to detection of acute myocardial injury and after the endovascular procedures in the remaining 7 (50%) patients. Ischemic ECG changes were not present in any of the 14 patients with elevated hsTnT. Out of the 14 patients with elevated hsTnT, nine (64.3%) received PCI. PCI was performed in all three coronary arteries in one patient, in two out of three in three patients, in a coronary graft in one patient and in one out of three arteries in the remaining four patients (mean number of stents 3.2 ± 2.4). 

Patients with elevated hsTnT more frequently underwent cardiac catheterization and PCI compared to those with normal hsTnT (85.7% versus 4.8% and 64.3% versus 1.8%, *p* < 0.001 for both).

### 3.5. Predictors of Cardiac Work-Up and PCI

**Figure 2:**
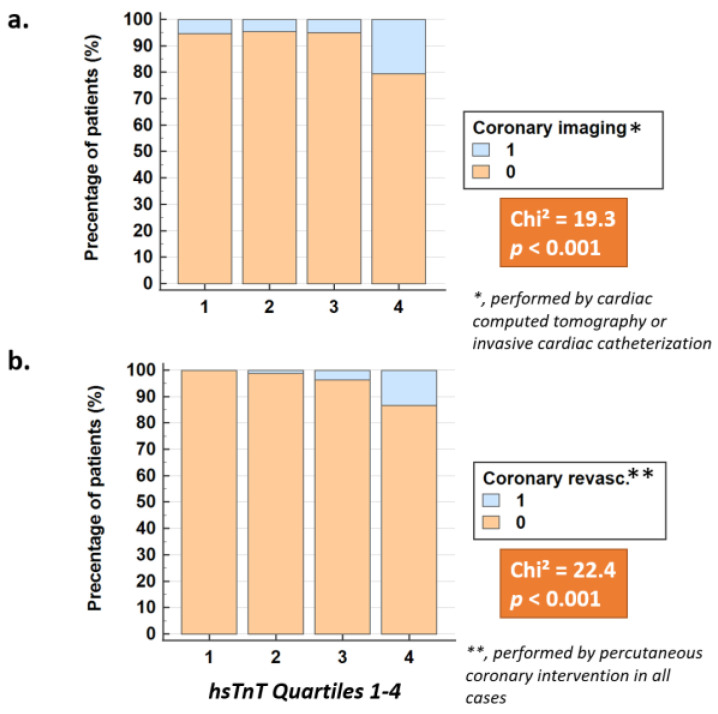
HsTnT quartiles were associated with subsequent coronary imaging by CCTA or invasive angiography (**a**) and with PCI (**b**) in patients with claudication (*p* < 0.001 for both).

Subsequent CCTA or invasive angiography (Figure 2a) and PCI procedures (Figure 2b) increased with increasing hsTnT quartiles (*p* < 0.001 for both). In addition, hsTnT values and hstnT quartiles were strong predictors of subsequent PCI by multivariable analysis, independent of age, cardiovascular risk factors and impaired kidney function (Table 2). 

### 3.6. Primary Endpoint and All-Cause Mortality 

**Figure 3:**
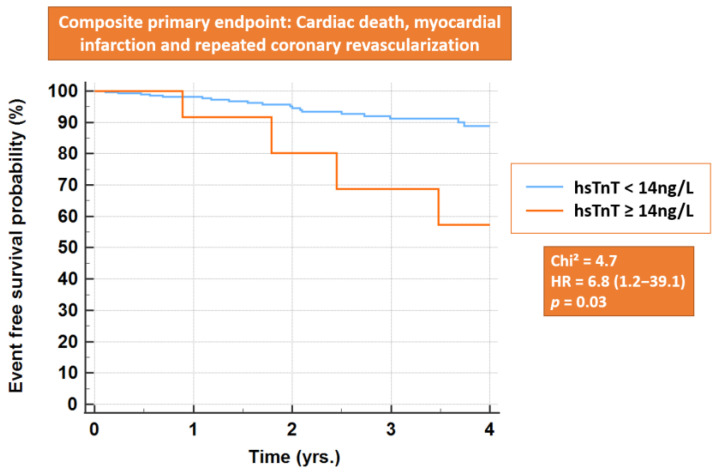
Kaplan-Maier analysis. Higher rates of cardiac events are present during follow-up in patients with elevated (n_elevated_ = 14) versus normal baseline hsTnT (n_normal_ = 332) (*p* = 0.03 by log-rank test).

Follow-up was complete in 299 (86.4%) patients. The mean follow-up duration was 2.4 ± 1.4 years (median 2.5 yrs, 95%CI = 2.1–2.7 yrs.). During follow-up, 20 of 286 (7.0%) patients with normal (one cardiac death, 10 myocardial infarctions and nine urgent coronary revascularizations) versus four of 13 (30.8%) with elevated hsTnT at baseline (one cardiac death, three coronary revascularizations) reached the composite primary endpoint. The corresponding Kaplan-Maier analysis confirmed a higher cardiac event rate in patients with elevated baseline hsTnT (Figure 3, *p* = 0.03 by log-rank test). 

Seven patients died during follow-up, all with normal baseline hsTnT values due to heart failure (*n* = 1), sepsis (*n* = 2), cancer (*n* = 3) and unknown causes (*n* = 1).

## 4. Discussion

This analysis reports on the role of cardiac troponins for the clinical management of patients referred for elective endovascular treatment due to claudication in the largest cohort so far, to the best of our knowledge. Four percent of patients with claudication (RC 2 and 3) and without cardiac symptoms exhibited increased cardiac troponins by hsTnT, including 2.0% with proof of acute myocardial injury by serial troponin sampling. A history of CAD and especially of myocardial infarction are strong independent predictors of elevated hsTnT in PAD patients. A cardiac diagnostic work-up seems justified in patients with elevated baseline hsTnT since PCI was subsequently performed in 9 of 14 (64.3%) of these patients. In addition, hsTnT may forecast long-term clinical outcomes in patients, including cardiac death, myocardial infarction, and repeated coronary revascularization. In patients with a history of CAD or myocardial infarction who present with claudication, hsTnT evaluation and extended cardiac work-up may be necessary since such patients with polyvascular disease may exhibit increased risk for CAD progression and acute myocardial injury and may profit from coronary revascularization and more aggressive conservative treatment. Therefore, assessing hsTnT in each patient with polyvascular disease may be useful for the risk stratification of such patients at very high risk for future cardiovascular events [24]. 

### 4.1. Previous Studies and Current Recommendations

Previous studies highlighted the usefulness of cardiac troponins for the risk stratification of patients with chronic coronary syndromes (CCS) and in the general population [8,9]. In both cohorts, the presence of cardiac troponins and especially hsTnT predicted cardiovascular complications precisely, so that these patients with elevated hs-TnT levels merit intense medical attention. Patients with PAD, on the other hand, are per se at high risk for future cardiovascular complications such as death, myocardial infarction, and stroke [25]. In the same direction, recent studies highlighted the increased risk of patients with poly-vascular atherosclerotic disease for future cardiac and limb events, stressing the essential role of disease burden quantification and the need for aggressive preventive strategies in high-risk patients [26]. In a recent study conducted in the UK, cardiovascular mortality accounted for one-third of deaths in patients with symptomatic PAD, with most of these patients currently undergoing endovascular rather than open surgery for PAD treatment [27]. In addition, the hospital costs incurred from the hospitalization of patients with limb ischemia were recently shown to be significantly higher even when compared to patients with stroke in a prospective, population-based study [28]. 

However, the need for further cardiac work-up in patients with symptomatic PAD but without cardiac symptoms remains controversial. Thus, routine cardiac diagnostic work-up by cardiac CT or catheterization in such patients may result in CAD overtreatment without necessarily translating to a clinical benefit, especially in cardiac asymptomatic patients, as recently demonstrated in the ISCHEMIA study [29]. Furthermore, a recent study indicated unfavorable clinical outcomes with higher repeated coronary revascularization rates after PCI in patients with PAD, possibly attributed to the higher incidence of diffuse CAD due to diabetes and renal failure. Out of 9204 patients who underwent PCI, 695 patients also had symptomatic PAD. PAD was associated with an increased risk for death, cardiovascular events and complications after PCI [30]. A recent meta-analysis of randomized trials, on the other hand, showed that cardiac outcomes and the rate of spontaneous infarctions during follow-up were improved in patients with CCS undergoing elective coronary revascularization and optimal medical treatment versus medical treatment alone [31]. Thus, although a high percentage of patients with symptomatic PAD have concomitant asymptomatic CAD, patient selection may be more complex than anticipated. 

In a recent meta-analysis, the role of cardiac troponins was investigated for the risk stratification of PAD patients. In this study, which included 5313 patients with symptomatic PAD, elevated baseline troponin values were significantly associated not only with major cardiovascular complications but also with an increased amputation rate during a median follow-up of 2.3 yrs [11]. Interestingly, baseline troponin was elevated in 5% of patients with claudication (like in our cohort) and in 63% of patients presenting with critical limb threatening ischemia (CLTI). In a study including 242 patients with PAD, hsTnT and lipoproteins were significantly increased in patients with critical limb threatening ischemia compared to those with claudication, indicating more advanced atherosclerotic disease in patients with CLTI [32]. Moreover, in another study, hsTnT was utilized to assess post operative myocardial injury in patients with CLTI undergoing endovascular treatment. According to the results, 85.2% of the patients with postoperative myocardial injury and elevated troponin levels did not exhibit either cardiac related symptoms orECG changes compared to the baseline preoperative assessment. The study is also indicative of the important role of cardiac troponins for the clinical management of such patients with PAD und underlying silent CAD [33]. Furthermore, a new cardiac diagnostic test, coronary computed tomography derived fractional flow reserve, was shown to identify silent underlying CAD in 69% of patients with CLTI who subsequently benefit from coronary revascularization in terms of cardiovascular endpoints such as repeated coronary revascularization, myocardial infarction, and survival during 3 years of follow-up [34,35].

### 4.2. Current Findings 

In our study cohort, baseline troponin values were elevated in ~4% of the patient cohort. Importantly, patients with acute limb ischemia or CLTI and RC 4-6, who per se undergo peripheral revascularization for limb salvage, exhibit increased frailty and in some cases limited life expectancy, so that additional elective cardiac interventions may be of limited clinical value, especially in older and frail patients. Therefore, we excluded these patients from the present analysis. However, such patients would also profit from more aggressive conservative management. In previous studies, up to >60% of patients with CLTI exhibited elevated cardiac troponins, which makes it difficult to stratify their risk by cardiac biomarkers. Importantly, hsTnT exhibited high precision for the risk stratification of patients with claudication in the present study. Thus, only 5.4% of patients with normal hsTnT < 14 ng/L underwent a further cardiac diagnostic work-up, and PCI was necessary in only 1.8% of cases. On the other hand, all patients with elevated hsTnT at baseline underwent cardiac diagnostic work-up by CCTA or invasive catheterization, and 64.3% required urgent or elective PCI. In addition, baseline hsTnT was related to cardiovascular risk factors and serum creatinine, whereas patients with increased hsTnT exhibited higher rates of cardiac events during follow-up, as shown by Kaplan-Maier analysis. Notably, all patients with increased hsTnT at baseline underwent extensive cardiac work-up. This was not always the case in most patients with normal baseline hsTnT, where cardiac work-up except for ECG was not routinely performed. Therefore, patients with elevated baseline hsTnT may have received increased medical attention and more aggressive lipid-lowering therapies during follow-up on top of prompt revascularization compared to other patients, all of which may have positively influenced the subsequent rate of cardiac events. Despite this consideration, cardiac events were higher at follow-up compared to the patient group with initially normal troponin values.

### 4.3. Limitations

Our study has some limitations. Firstly, the number of patients included in the present study derives from a single center and is therefore relatively small. In the same direction, the number of cardiac adverse events during follow-up was relatively small. In addition, hsTnT was measured in all patients presenting with symptomatic PAD without randomization, and the subsequent clinical management, as decided by the treating physicians, may have been biased by the baseline hsTnT value. However, troponin assessment should represent a standard measure in patients with elevated atherosclerotic risk, such as patients with symptomatic PAD, especially in those with a history of CAD and myocardial infarction. Furthermore, CCTA has been available in our institution since 2020, so that patients between 2018–2019 may have undergone cardiac catheterization more frequently than CCTA, compared to the patients thereafter. Furthermore, follow-up was not available in all our patients and both our study setting and the low number of cardiac events during follow-up do not allow for definitive conclusions on the influence of the baseline hsTnT measure on patient outcomes. This needs to be evaluated in future larger prospective studies.

In addition, patients with CLTI RC4-6 were intentionally excluded from analysis. Such patients were previously reported to frequently exhibit increased cardiac troponins [11,21], possibly triggered by pre-existing advanced coronary artery disease, heart failure with increased myocardial wall tension, impaired renal clearance or by increased inflammation due to extensive wound healing disorders and sepsis [11].

Finally, genetic samples based on whole blood RNA sequencing were not included in our analysis, which is a limitation. The investigation of such genotypes in patients with CAD and PAD, together with biochemical markers such as cardiac troponins, merits further investigation in future studies.

## 5. Conclusions

In summary, our study highlights that patients with claudication are at high cardiovascular risk, since 4% of them exhibit an elevated hsTnT, including 2% with acute myocardial injury. Specifically, patients with a history of CAD and myocardial infarction exhibit a high risk for hsTnT elevation although being cardiac asymptomatic. In such patients, further cardiac diagnostic work-up may be required on a routine basis, since in some cases this may trigger urgent coronary revascularizations, which needs to be performed prior to the endovascular revascularization procedure for PAD treatment. In addition, an aggressive therapeutic strategy is pivotal for the prognosis of patients with high-risk polyvascular disease.

## Figures and Tables

**Table 1:** Demographic data, laboratory markers and cardiac medications of patients with claudication and normal versus elevated baseline troponin values.

Baseline and Laboratory Data	All Patients (*n* = 346)	Patients with Normal hsTnT (*n* = 332)	Patients with Elevated hsTnT (*n* = 14)	*p*-Values
	Demographic Data			
Age	68.3 ± 9.5	68.2 ± 9.4	69.1 ± 12.0	0.72
Female gender	140(40.5%)	138(41.6%)	2(14.3%)	0.04
1. Arterial hypertension	318(91.9%)	304(91.6%)	14(100%)	0.26
2. Hyperlipidemia	274(79.2%)	261(78.6%)	13(92.9%)	0.42
3. Diabetes mellitus	98(28.3%)	91(27.4%)	7(50.0%)	0.07
4. Active or former smoking	232(67.1%)	221(66.5%)	11(84.6%)	0.47
Total number of risk factors (0–4)	2.7 ± 0.9	2.6 ± 0.9	3.2 ± 0.7	0.03
History of CAD	108(31.2%)	98(29.5%)	10(71.4%)	0.001
History of myocardial infarction	36(10.4%)	27(8.1%)	9(64.3%)	<0.0001
History of PCI	33(9.5%)	30(9.0%)	3(21.4%)	0.12
History of CABG	28(8.1%)	24(7.2%)	4(28.6%)	0.0001
	Cardiac Medications			
Aspirin	339(98.0%)	328(98.8%)	11(78.6%)	0.43
Dual platelet therapy after treatment	285(82.4%)	274(82.5%)	11(78.6%)	0.70
Oral anticoagulants	86(24.9%)	83(25.0%)	3(21.4%)	0.73
Diuretics	112(32.3%)	105(31.6%)	7(50.0%)	0.15
ACE inhibitors or AT2 blockers	127(36.7%)	123(37.0%)	4(28.6%)	0.51
ß-Blockers	156(45.1%)	147(44.2%)	9(64.3%)	0.14
Statins	320(92.5%)	308(92.8%)	12(85.7%)	0.32
	Laboratory Data			
Estimated GFR(mL/min/1.73 m^2^)	76.2 ± 24.0	76.2 ± 23.7	74.1 ± 31.0	0.74
Creatinine(mg/dL)	0.93 ± 0.24	0.93 ± 0.23	1.08 ± 0.38	0.02
Urea(mg/dL)	34.3 ± 12.8	33.9 ± 12.2	44.2 ± 21.1	0.003
Hemoglobin(mg/dL)	14.2 ± 1.5	14.2 ± 1.5	13.9 ± 1.6	0.53
High-sensitive troponin T(ng/L)	11.1 ± 23.8	8.2 ± 3.1	80.1 ± 97.1	<0.001
High-sensitive troponin T(ng/L) (median and interquartile range)	8.4(5.8–11.2)	8.2(5.7–11.0)	45.3 (32.1–63.5)	<0.001
	Rutherford Categories (RC)			
RC 2	6(1.7%)	6(1.8%)	0(0%)	0.61
RC 3	340(98.2%)	326(98.2%)	14(100%)	0.61

CAD; coronary artery disease, PCI; percutaneous coronary intervention, CABG; coronary aortic bypass surgery and GFR; glomerular filtration rate, AT; Angiotensin, ACE; Angiotensin converting enzyme.

**Table 2:** Multivariable regression analysis regarding elevated levels of hsTnT and predictors of PCI.

Covariates	Coefficient	SE	Wald	Odds Ratios	95%Cl	*p*-Values
Predictors of elevated hsTnT including myocardial infarction as covariate						
Age	0.0289	0.040	0.51	1.02	0.95 to 1.11	0.47
Female gender	−2.079	0.84	6.01	0.12	0.02 to 0.65	0.01
Estimated GFR (ml/min/1.73 m^2^)	0.012	0.015	0.58	1.01	0.98 to 1.04	0.44
History of myocardial infarction	3.50	0.66	27.62	33.19	8.99 to 122.53	<0.0001
Predictors of elevated hsTnT including history of coronary artery disease as covariate						
Age	−0.0021	0.039	0.002	0.99	0.92 to 1.07	0.95
Female gender	−1.42	0.78	3.25	0.24	0.05 to 1.12	0.07
Estimated GFR (ml/min/1.73 m^2^)	−0.0033	0.014	0.052	0.99	0.96 to 1.02	0.81
History of coronary artery disease	1.75	0.62	7.95	5.8	1.71 to 19.74	0.005
Predictors of PCI including High-sensitive troponin T quartiles as a covariate						
Age	0.00119	0.032	0.001	1.00	0.93 to 1.06	0.97
Total number of atherogenic risk factors	0.688	0.32	4.4	1.98	1.05 to 3.76	0.03
Estimated GFR (ml/min/1.73 m^2^)	−0.00133	0.012	0.01	0.99	0.97 to 1.02	0.91
HsTnT (ng/L) quartiles	1.555	0.42	13.1	4.73	2.04 to 10.96	0.0003
Predictors of PCI including High-sensitive troponin T(ng/L) levels quartiles as a covariate						
Age	0.0417	0.04	1.06	1.04	0.96 to 1.12	0.30
Total number of atherogenic risk factors	0.748	0.36	4.31	2.11	1.04 to 4.27	0.03
Estimated GFR (ml/min/1.73 m^2^)	0.0021	0.014	0.021	1.00	0.97 to 1.03	0.88
HsTnT (ng/L)	0.064	0.016	14.58	1.06	1.03 to 1.1	0.0001

HsTnT: High-sensitive troponin T, PCI: percutaneous coronary intervention, and GFR: glomerular filtration rate.

## Data Availability

The dataset used and/or analyzed is available from the corresponding author upon reasonable request.

## Abbreviations

BTK	Below-the-knee
CAD	Coronary artery disease
CCS	Chronic coronary syndromes
CCTA	Cardiac computed tomography angiography
CFA	Common femoral artery
CLTI	Chronic limb-threatening ischemia
DCB	Drug coated balloon
DM	Diabetes mellitus
FFR	Fractional flow reserve
GFR	Glomerular filtration rate
HsTnT	High-sensitive troponin T
PAD	Peripheral arterial disease
PCI	Percutaneous coronary intervention
POBA	Plain old balloon angioplasty
RC	Rutherford category
